# Editorial: The functional role of non-coding RNAs in tumor microenvironment and metastasis of genitourinary tumor and its potential application as tumor molecular biomarkers

**DOI:** 10.3389/fgene.2023.1133496

**Published:** 2023-01-18

**Authors:** Zhongbao Zhou, Yuanshan Cui, Yong Zhang, Jitao Wu

**Affiliations:** ^1^ Department of Urology, Beijing TianTan Hospital, Capital Medical University, Beijing, China; ^2^ Department of Urology, The Affiliated Yantai Yuhuangding Hospital, Qingdao University, Yantai, China

**Keywords:** genitourinary tumor, metastasis, non-coding RNAs, therapeutic target, immune escape, biomarker

Genitourinary tumor was one of the most frequent malignant tumors in the world and has received a lot of attention due to its high incidence and fatality rates (Miller et al., 2022). The tumor microenvironment (TME) consists of immune cells, fibroblasts, lymphocytes, and other components that influence tumor growth (Dunn et al., 2004). Changes in the components of TME have a major impact on genitourinary tumor metastasis and lead to poor clinical outcomes. More and more non-coding RNAs (ncRNAs) including microRNAs (miRNAs), long non-coding RNAs (lncRNAs) and circular RNAs (circRNAs), are implicated in TME-mediated carcinogenesis, angiogenesis, invasion, metastasis and chemoresistance (Liu et al., 2021; Jiang et al., 2022; Rajtmajerová et al., 2022; Xu et al., 2022). Clarifying the regulatory link between ncRNAs and TME might aid in the development of innovative therapeutic options for genitourinary malignancies, which would be extremely advantageous in improving the prognosis of patients. The TME is complicated, and numerous ncRNAs are known to have an influence in the TME, but further research is needed to determine the particular regulatory functions (Shao et al., 2022). Furthermore, it is critical to understand how to control the abnormal expression of ncRNAs in the TME. As a result, we aimed to identify the crucial regulatory roles of ncRNAs in diverse components of the TME and to offer the theoretical foundation for their use in tumor diagnostics and therapy.

Impressively, we have chosen five outstanding publications from a large number of articles for this Research Topic. Four of them offered biomarkers to predict clinical outcomes, while another examined the connection between ncRNA and autophagy and its significance in bladder cancer (BC). We expect that these findings will shed fresh light on the features of TME as well as the treatment and prognosis of genitourinary malignancies.

Ferroptosis is a promising anticancer therapeutic target, and lncRNAs can influence ferroptosis *via* modulating related genes. aimed to identify the regulatory function of lncRNAs in ferroptosis and establish a theoretical foundation for their use in clear cell renal cell carcinoma (ccRCC). They constructed an ferroptosis-related lncRNAs model (including LINC00894, AL139123.1, ASMTL-AS1, AL157392.4, AL031714.1, AC135050.3, AP006621.2, NARF-IT1, YEATS2-AS1, LINC02804, AC024361.3, KIF1C-AS1, PCED1B-AS1, UBE2Q1-AS1, AL031705.1, AC005306.1, PTOV1-AS2, AC114730.3, AC073487.1, AC104564.3, AC020907.4, AC005387.2, AL513218.1, and AC025766.1), and reported that this model played a significant role in the prognosis and immune microenvironment of ccRCC patients.

N6-methyladenosine (m6A) is the most prevalent type of ncRNA-specific internal modification seen in eukaryotes, and it has a significant impact on mRNA stabilization, translations, and splice sought to evaluate five m6A-lncRNAs model (including NFIA-AS2, NR2F1-AS1, MIR99AHG, TMEM147-AS1, RAP2C-AS1) of BC patients, and discovered that this model was important in predicting the effect of immunotherapy and prognosis, as well as demonstrating its potential biological function through pathway enrichment.

Pyroptosis is a host cell death mechanism triggered by a variety of microbial infections and non-infectious stimuli, with caspase-1 reliance being a distinguishing feature. Due to the tight association between pyroptosis and human cancer reported in a vast number of publications used adequate bioinformatics analysis and initial RT-qPCR confirmation *in vitro* to build a predictive pyroptosis-related lncRNAs (PRLs) model (including OCIAD1-AS1, MAFG-DT, SLC25A25-AS1, SNHG18, PSMB8-AS1, TRIM31-AS1) and two ceRNA pathways. The findings suggested that PRLs played a crucial role in immune cell infiltration and anti-tumor drug sensitivity in BC patients, and that regulatory axis OCIAD1-AS1/miR-141-3p/GPM6B and OCIAD1-AS1/miR-200a-3p/AKAP11 may perform a contribution to the growth of BC.

Together with progress of RNA-sequencing, miRNA editing events have been shown to play a key role in many cancers examined the mRNA and miRNA transcriptomes of 12 recurrent BC patients and 13 primary BC patients to identify and compare miRNA editing events. The research revealed that miR-154-5p was highly altered in recurrent BC and was related to patient prognosis, implying that A-to-I editing of miR-154-5p might be a viable target for BC therapy. Additionally, miR-154 editing events may be important in the categorization of BC consensus molecular subtypes, allowing existing therapy options to be broadened and personalized.

Lastly highlighted recent results on ncRNAs and its association with numerous physiological functions such as autophagy, as well as its relevance to the pathophysiology of BC. Several researches already have proven the role of ncRNAs in modulating metastatic capability and deliberating medication sensitivity in BC. The particular functions of ncRNA in BC growth and their clinical importance, however, are still being researched. The importance of ncRNA would be described in full using new cutting-edge technologies, and prospective translational usefulness for BC patients’ management will be demonstrated. Additionally, targeting both ncRNAs and autophagy simultaneously may be a promising treatment strategy for BC.

This Research Topic and existing research of ncRNAs in genitourinary tumor still have various limitations. First, many studies in this Research Topic are just the re-analysis of public databases, lacking the validation of relevant cell experiments and clinical samples, which makes the conclusions need to be carefully considered. Second, as compared to mRNAs, lncRNAs are often poorly conserved across species. Therapeutic techniques based on cellular and animal models are still far from clinical applicability and may need more study. Finally, the enrichment mechanism of ncRNAs in tumor phenotype and immunological invasion features has to be confirmed. Many recent investigations are still in the theoretical stage due to the aforementioned restrictions. These three characteristics should be given more consideration in future research.
